# Predicting freeway incident duration using machine learning

**DOI:** 10.1371/journal.pone.0359353

**Published:** 2026-09-30

**Authors:** Yanni Ju, Jingqi Liu, Mostafa K. Ardakani, Wanqiu Li, Gen Li

**Affiliations:** 1 Key Laboratory of Intelligent Policing and National Security Risk Management, Sichuan Police College, Luzhou, China; 2 Department of Road Traffic Management, Sichuan Police College, Luzhou, China; 3 Intelligent Policing Key Laboratory of Sichuan Province, Luzhou, China; 4 College of Automobile and Traffic Engineering, Nanjing Forestry University, Nanjing, Jiangsu, China; 5 Department of Information Systems and Business Analytics, Kent State University, Ohio, United States of America; 6 Key Laboratory of Automotive Measurement, Control and Safety, Xihua University, Chengdu, China; Villanova University, UNITED STATES OF AMERICA

## Abstract

Accurate prediction of freeway incident duration is important for traffic management and emergency resource allocation. This study evaluates 32 machine-learning model configurations using 828 incident records from the G2504 Hangzhou Ring Freeway in China. Four fixed feature sets containing the top 6, 11, 16, and 21 predictors were defined from a Minimum Redundancy Maximum Relevance (MRMR) ranking. Each feature setting was evaluated using a 15% hold-out test set, seven repeated random splits, and sevenfold cross-validation within the training subset. All 32 machine-learning models were tuned within the training subset using sevenfold cross-validation. To provide a concise description of the tuning procedure, the selected Medium Gaussian support vector machine, which provided the most balanced overall performance, is reported as a representative example. Performance was assessed using the mean absolute error (MAE) and the mean absolute percentage error (MAPE). The Medium Gaussian support vector machine provided the most balanced overall performance, and prediction accuracy generally improved from 6 to 16 predictors, with little additional benefit from 21 predictors. Compared with classical linear regression, the Medium Gaussian support vector machine reduced MAE by 5.7%–9.7% and MAPE by 21.2%–31.9% across the four feature settings. A sensitivity comparison showed substantially higher errors when incidents longer than 60 minutes were retained, reinforcing that the main findings apply to routine incidents. Interpretation analyses identified Response Time and severity-related variables as important predictors, while residual errors remained larger for longer and more complex incidents. The model is intended primarily for post-arrival updating and clearance-support decisions for routine freeway incidents, rather than prediction at the initial alarm stage.

## Introduction

Freeways carry heavy traffic at high speeds, so even minor incidents can cause substantial disruption if they are not resolved quickly. Delayed clearance may trigger secondary crashes, worsen congestion, and increase overall travel delays, making efficient incident management a critical component of freeway operations. Effective traffic control during an incident, such as dynamic lane management and timely travel information provision, is also essential for maintaining stable traffic flow until normal conditions are restored. While minor incidents are often cleared within a short time through routine on-site actions, more severe events, including rollovers, hazardous material spills, heavy-truck crashes, and casualty-related accidents, may require multi-agency coordination, specialized equipment, and extensive safety inspections, resulting in much longer clearance times [1]. Accurate prediction of freeway incident duration is therefore important for emergency dispatch, congestion mitigation, and overall traffic management efficiency.

Over the past few decades, researchers have explored a variety of methods for incident duration prediction. Previous studies have shown that incident duration is influenced by multiple factors, including incident characteristics, vehicle involvement, injury severity, and environmental conditions [2]. Traditional statistical approaches, such as hazard-based duration models, provide strong interpretability because they help explain how specific factors affect incident clearance times. However, these approaches often rely on restrictive assumptions and may not adequately capture complex and nonlinear relationships. In contrast, machine-learning methods have demonstrated strong potential for improving predictive performance in incident duration prediction [3]. Recent reviews have highlighted the importance of systematic comparison and predictive robustness, while explainable AI studies have emphasized interpretability [4]. The present study is positioned as an application-oriented configuration-level comparison rather than as a new machine-learning framework. Its contribution is threefold: first, 32 candidate model configurations are compared under a common repeated hold-out framework; second, four fixed MRMR-ranked feature sets are used to examine the trade-off between predictive performance and model simplicity; and third, Shapley importance, partial-dependence analysis, validation plots, and residual diagnostics are combined to identify the conditions under which prediction errors remain large.

The remainder of this paper is organized as follows. The next section reviews the relevant literature. The data and methodology are then described, followed by the results, discussion, and policy implications, and conclusions.

### Literature review

Accurate prediction of traffic incident duration is fundamental to traffic operations and incident management because duration directly affects congestion propagation, emergency response efficiency, resource deployment, and network recovery. Existing review studies have shown that incident duration prediction has evolved from relatively simple statistical estimation into a mature research area involving multiple data sources, diverse modeling paradigms, and increasing attention to operational applicability [5,6]. At the same time, studies on non-recurrent congestion, traffic mobility disruption, and spatial characteristics of traffic incidents have consistently shown that longer incidents are associated with broader traffic impacts and greater management difficulty [7–9]. These findings confirm that incident duration is not only a descriptive indicator of incident severity, but also a practical variable that shapes traffic control decisions and post-incident recovery strategies.

Early traffic incident duration studies mainly relied on statistical duration-analysis methods. Hazard-based and survival-analysis models established the methodological foundation for examining incident clearance processes and identifying significant contributing factors [10,11]. Later studies extended this tradition by introducing competing-risk formulations and joint modeling frameworks to address sequential incident stages and related operational dependencies [12,13]. More recently, causal and heterogeneity-oriented statistical approaches have further emphasized that incident duration is influenced by complex interactions, unobserved heterogeneity, and context-specific mechanisms [14]. Although these approaches remain valuable because of their interpretability, review papers have repeatedly noted their limitations in dealing with nonlinear effects, mixed data structures, and high-dimensional predictor spaces. For this reason, the literature has gradually shifted toward more flexible machine-learning approaches.

Machine-learning methods are now widely used in incident duration prediction because of their capacity to capture nonlinear relationships and interactions among incident, roadway, environmental, and response-related variables. Earlier studies demonstrated the usefulness of tree-based and rule-based approaches, such as M5P, for freeway lane-clearance prediction [15]. Subsequent research expanded toward random forests, support vector machines, gradient boosting, and adaptive feature-set strategies [16–20]. For example, Tang et al. showed that XGBoost can effectively support both prediction and influencing-factor analysis in incident clearance time studies [17], while Ghosh et al. demonstrated the value of adaptive feature sets for dynamic prediction [18]. Li et al. further highlighted that sequential prediction is especially important for large-scale incidents, where information becomes available gradually over time [19]. Grigorev et al. proposed a bi-level machine-learning framework with outlier removal and intra–extra joint optimization, illustrating the growing interest in more structured prediction pipelines rather than one-step black-box comparison alone [20]. Overall, this line of research suggests that the field is moving from single-model applications toward broader comparative evaluation and more refined prediction frameworks.

In parallel, recent studies have increasingly explored deep learning, text mining, multimodal fusion, and explainable artificial intelligence in traffic incident and safety prediction. Hybrid deep-learning methods based on multi-source incomplete data have been used to improve prediction under complex and partially observed traffic conditions [21]. Text mining and ensemble learning have also been introduced to exploit textual incident descriptions, showing that narrative information can improve prediction performance when structured variables are limited [22]. More recently, multimodal data and ensemble deep-learning architectures have been used to combine heterogeneous inputs, while generative models and transformer-based methods have been proposed to address data imbalance and improve performance for more difficult prediction cases [23,24]. Ensemble-learning studies have likewise shown that heterogeneous model combinations may outperform single learners in some contexts [25]. Recent vision-based crash-detection research has further emphasized the importance of maintaining prediction consistency across complex traffic scenes, such as day–night conditions, through depth-aware and domain-adaptive network designs [26]. At the same time, explainable machine learning has become increasingly important. Hamad et al. applied XGBoost and SHAP to incident duration analysis and showed that prediction and explanation can be integrated in a practically meaningful way [4]. These studies indicate that the current frontier of incident-related prediction research is no longer limited to prediction accuracy alone, but increasingly concerns interpretability, robustness, and deployment relevance.

Beyond the incident-duration literature itself, broader transportation safety and operations research also provides methodological insights relevant to this topic. Li et al. developed a correlated random-parameter duration framework for traffic conflict interval time and highlighted the importance of modeling heterogeneity in duration-related processes [27]. Yang et al. reviewed resilience assessment and enhancement in urban integrated transportation networks, emphasizing the need to connect predictive analytics with system robustness and operational management [28]. In a related safety-analysis context, Li et al. used Light Gradient Boosting Machine and Shapley additive explanations to identify factors associated with E-bike rider hit-and-run behavior, illustrating the value of interpretable machine learning in transportation safety studies [29]. Luo et al. further proposed a real-time early-warning framework for multidimensional heavy-duty truck driving risk using trajectory data, showing how predictive models can be integrated into real-time management systems [30]. Recent freeway-merging research has also used spatiotemporal frameworks to predict conflict evolution and hotspot migration under connected and automated vehicle mixed-traffic conditions, indicating that dynamic risk representation is becoming increasingly important in freeway safety analysis [31,32]. Although these studies are not all direct incident-duration studies, they collectively indicate a broader methodological trend in transportation research toward combining predictive performance, explainability, robustness, and operational utility.

Despite this progress, several gaps remain. First, many studies compare only a small number of models or rely on a single data split, making reported rankings sensitive to the evaluation design. Second, the effect of feature dimensionality has not often been examined across a broad set of candidate configurations. Third, fewer studies combine configuration-level comparison, feature reduction, global explanation, category-level validation, and residual diagnosis within one workflow. The present study therefore evaluates 32 machine-learning model configurations under four fixed feature sets, while explicitly limiting its claims to the current data and validation framework.

## Materials and Methods

### Data

This study uses traffic incident data from the G2504 Hangzhou Ring Freeway (Hangjin–Quzhou section) in Zhejiang Province, China, covering the full year of 2021. The original dataset contained 1,066 incident records. Records with missing critical time variables were first removed, leaving a complete-case dataset of 911 incidents before duration filtering. Incidents lasting more than 60 minutes were then excluded from the main modeling sample, resulting in 828 routine incidents. No missing-value imputation was performed. The 60-minute threshold was used to focus the primary analysis on routine incidents because prolonged incidents may involve extended towing, secondary-event handling, long lane closures, multi-agency coordination, or on-site investigation processes that were not fully represented by the available structured predictors. The main results should therefore be interpreted as applying primarily to incidents lasting 60 minutes or less. Categorical predictors were represented through automatic full dummy encoding (one-hot encoding) during model training.

Table 1 summarizes the 21 candidate predictors, including one continuous variable and 20 categorical variables. Response Time is the interval, in minutes, between alarm activation and first-responder arrival; because it becomes known only after responders arrive, models that include this variable support post-arrival updating rather than initial-alarm prediction. Incident Duration is the elapsed time from incident occurrence to final clearance. Four fixed feature sets containing the top 6, 11, 16, and 21 predictors were defined from the MRMR ranking. The Death variable was retained for consistency with the original data schema, but it had zero variance in the filtered sample and therefore provided no discriminatory information. Several source variables used reverse coding, including Vehicle Breakdown (0 = breakdown, 1 = no breakdown) and Incident Severity (0 = serious incident, 1 = other); these coding directions were retained and considered when interpreting model effects.

**Table 1:** Independent Variable Descriptions.

No.	Variable	Description	Levels {Number of observations out of 828}
1	Accident Type	Type of traffic accident	0 = Rear-end {603}, 1 = Object {169}, 2 = Side {46}, 3 = Rollover {4}, 4 = Fire {6}
2	Time of Day	Time period when the accident occurred	0 = Daytime {45}, 1 = AM Peak {445}, 2 = PM Peak {115}, 3 = Nighttime {223}
3	Holiday	Whether the accident occurred on a public holiday	0 = No {731}, 1 = Yes {97}
4	Weekend	Whether the accident occurred on a weekend	0 = No{564}, 1 = Yes{264}
5	Section	Road section where accident occurred	0 = Hongken–Hongken Hub{135}, 1 = Hongken Hub–Xinjie{60}, 2 = Keqiao–Zhangjiafan{166}, 3 = Xiaoshan–Keqiao West{266}, 4 = Xinjie–Xiaoshan East{201}
6	Location	Location classification of accident site	0 = On-road {734}, 1 = Bridge {30}, 2 = Service Area {60}, 3 = Toll Station {4}
7	Alarm Source	Source of accident alarm/report	0 = Video surveillance {476}, 1 = Telephone report {302}, 2 = Manual patrol {50}
8	Weather	Weather conditions during the accident	0 = Sunny {380}, 1 = Rainy {158}, 2 = Foggy/Snowy {290}
9	Number of Vehicles	Number of vehicles involved	0 = Single-vehicle{166}, 1 = Two-vehicle{546}, 2 = Multi-vehicle{116}
10	Vehicle Breakdown	Whether there was a vehicle breakdown	0 = Breakdown{290}, 1 = No breakdown{538}
11	Injury Involvement	Whether the accident caused any injuries	0 = No Injury {804}, 1 = At least one injury {24}
12	Death	Whether the accident involved fatalities	0 = No deaths {828}, 1 = At least one death {0}
13	PC	Whether a private car was involved	0 = No{105}, 1 = Yes{723}
14	HV	Whether a heavy vehicle was involved	0 = No{591}, 1 = Yes{237}
15	Incident Severity	Whether the accident was classified as serious	0 = Serious incident {24}, 1 = Others {804}
16	Lane 1 Blocked	Whether lane 1 was blocked	0 = No{648}, 1 = Yes{180}
17	Lane 2 Blocked	Whether lane 2 was blocked	0 = No{751}, 1 = Yes{77}
18	Lane 3 Blocked	Whether lane 3 was blocked	0 = No{748}, 1 = Yes{80}
19	Lane 4 Blocked	Whether lane 4 was blocked	0 = No{519}, 1 = Yes{309}
20	Hard Shoulder Blocked	Whether the hard shoulder was blocked	0 = No{243}, 1 = Yes{585}
21	Response Time	Time from incident alarm/report to first responder arrival	Continuous variable [1,30]{828}

Fig 1 presents the relationship between response time and incident duration. The marginal distributions indicate that both variables are right-skewed. Response time is concentrated at lower values, whereas incident duration also shows a skewed distribution. The scatterplot shows a positive association between the two variables. The Spearman correlation coefficient is 0.51 (p < 0.001), indicating that incidents with longer response times tend to have longer overall durations.

**Fig 1:**
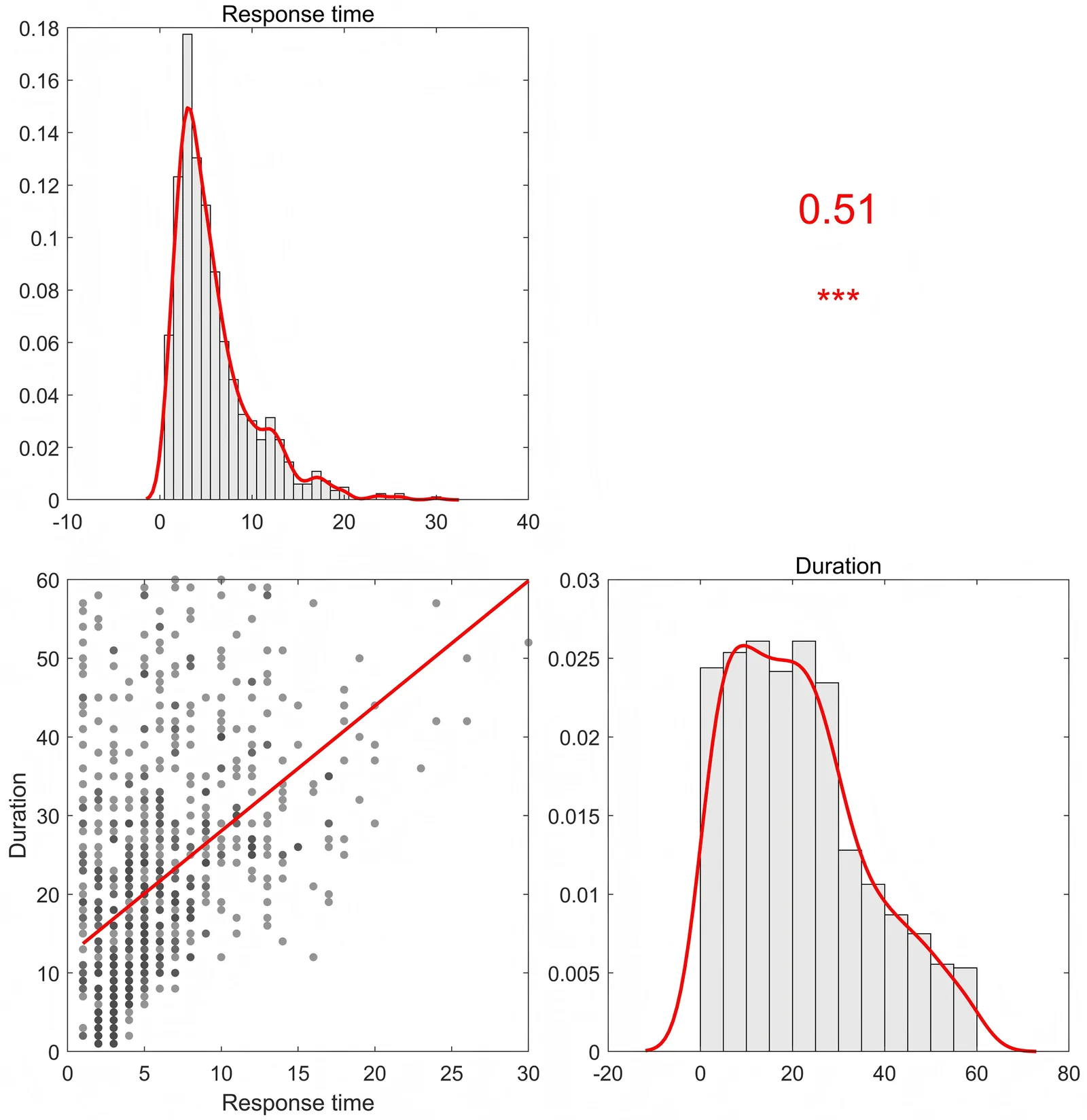
Response time and incident duration: marginal distributions and correlation.

### Methodology

To predict incident duration, the study evaluated 32 candidate model configurations under a fixed workflow. First, the complete-case and routine-incident samples were defined. Second, the four fixed MRMR-ranked feature sets (F6, F11, F16, and F21) were specified before the hold-out results were examined and were kept unchanged across all seven runs. Third, each feature set was evaluated using seven repeated random 85%/15% splits, with mutually exclusive training and hold-out subsets within each split. Fourth, one-hot encoding, model fitting, cross-validation, and hyperparameter selection were conducted using the training subset. Finally, the hold-out subset was reserved exclusively for final performance evaluation and was not used for model fitting or hyperparameter selection. The MRMR ranking was calculated once using the complete filtered sample before repeated splitting and was not re-estimated within each training subset. Consequently, observations later assigned to the hold-out subsets contributed to the initial feature ranking, so some feature-selection optimism cannot be excluded.

All 32 machine-learning models were tuned within the training subset using sevenfold cross-validation. Hyperparameter settings were selected separately for each model and repeated split, while the hold-out test subset was reserved exclusively for final performance evaluation. Because reporting the complete model-specific tuning records for all 32 models would be extensive, the tuning procedure for the selected Medium Gaussian SVM, which provided the most balanced overall performance, is reported as a representative example. For the Medium Gaussian SVM, Bayesian optimization was used to search for BoxConstraint, KernelScale, and Epsilon. The numerical search bounds were generated automatically by MATLAB Regression Learner from the training data and were not manually restricted. The optimization was conducted for 30 objective evaluations, using sevenfold cross-validated mean squared error as the model-selection criterion. As shown in Fig 2, the optimal model used standardized predictors and had BoxConstraint = 121.2512, KernelScale = 8.5858, and Epsilon = 0.1344. The observed minimum cross-validated MSE was 133.4013.

**Fig 2:**
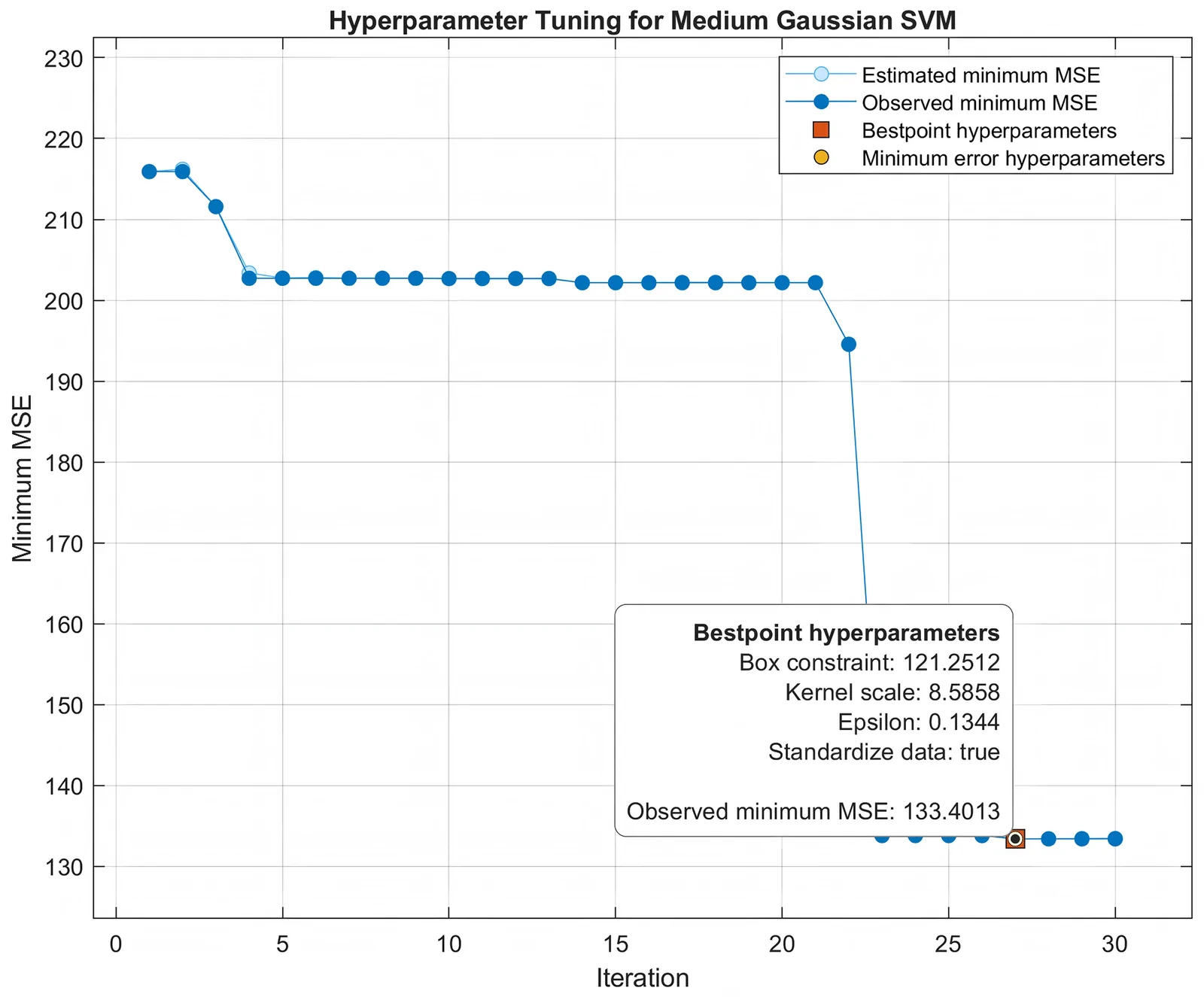
Example of SVM hyperparameter optimization in MATLAB Regression Learner.

For all feature configurations, seven repeated random splits and sevenfold cross-validation within the training subset were used. The evaluation framework therefore produced 896 model evaluations (4 feature sets × 32 models × 7 runs). Performance on the hold-out subset was summarized using median and interquartile range (IQR) values for MAE and MAPE. For the F16 model comparison, each model’s test MAE and MAPE were averaged across the seven repeated runs, and the corresponding two-sided 95% confidence intervals for both metrics were estimated using the t distribution with six degrees of freedom. Incident durations in the routine sample ranged from 1 to 60 minutes and contained no zero values; nevertheless, 123 incidents lasted 5 minutes or less, so MAPE remains sensitive to short-duration observations and is interpreted together with MAE rather than in isolation. Interpretation analyses were conducted using the saved tuned F16R2 Medium Gaussian SVM model because its test MAPE (67.87%) equaled the seven-run median for the F16 Medium Gaussian SVM. The same F16R2 model was used for the Shapley, partial-dependence, residual, and category-level validation analyses. Global Shapley importance was calculated using 200 query points. All computations were conducted using MATLAB R2024b and the corresponding Python implementations used for the evaluated models.

## Results

### Feature Selection

The MRMR method was used to rank the candidate predictors according to their relevance to incident duration while reducing redundancy among variables. Fig 3 presents the resulting importance scores. Response Time had the highest MRMR importance score, followed by Injury Involvement and Hard Shoulder Blocked. Accident Type, Alarm Source, Weekend, HV involvement, Time of Day, and Incident Severity also provided meaningful predictive information, whereas the remaining variables had comparatively smaller scores. This pattern suggests that operational and severity-related factors account for a substantial proportion of the predictive signal in the current dataset.

**Fig 3:**
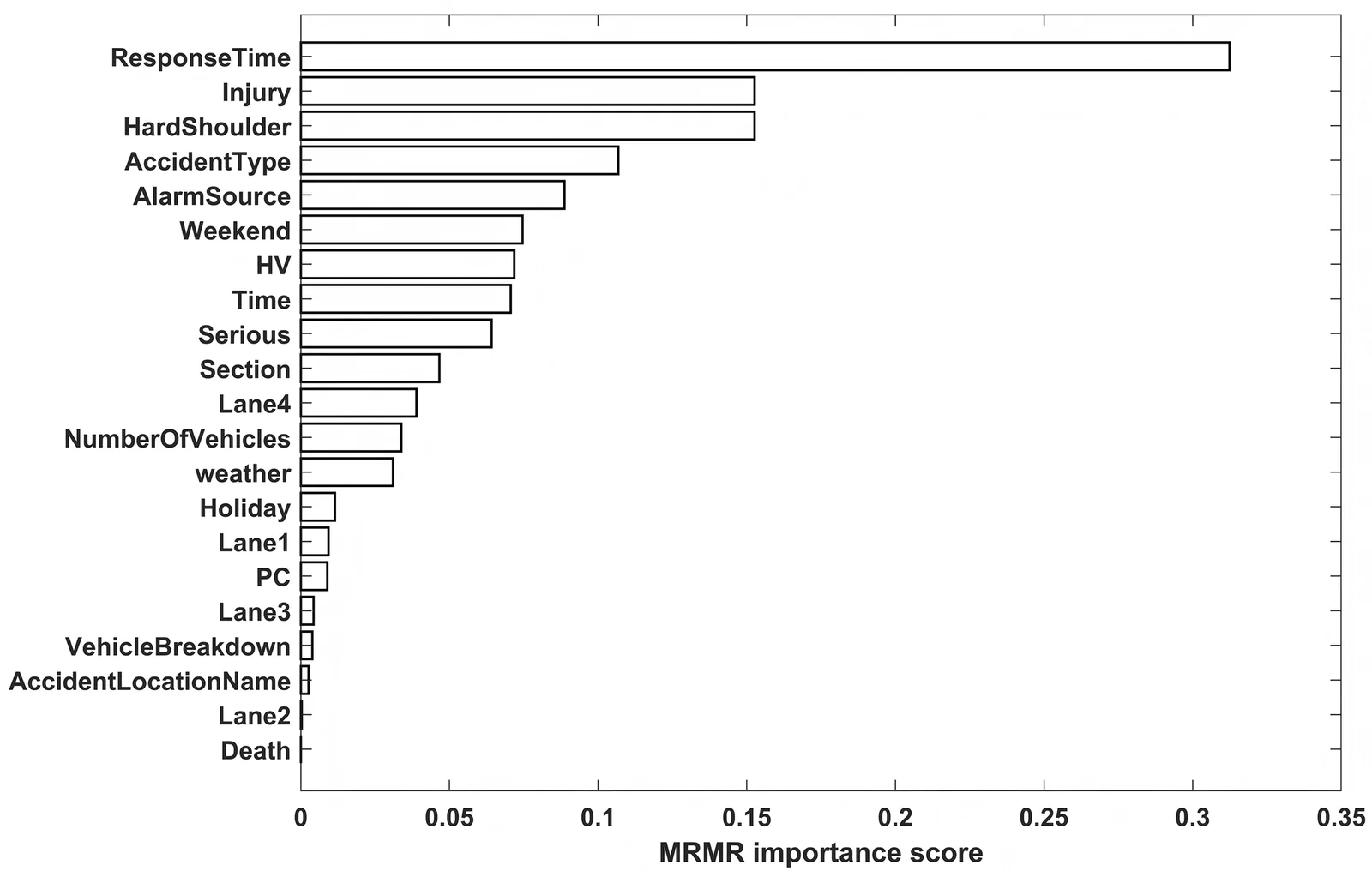
MRMR importance scores of the candidate predictors.

Based on the MRMR ranking, four feature sets containing the top 6, 11, 16, and 21 predictors were constructed for subsequent model comparison. These feature sets were used to examine how predictive performance changes as additional variables are introduced. The ranking pattern indicates that a relatively compact set of predictors may already capture most of the useful information for incident duration prediction, whereas the incremental contribution of lower-ranked variables appears to be smaller.

### Performance Comparisons

Tables 2 and 3 summarize hold-out performance for the 32 tuned model configurations across F6, F11, F16, and F21. Table 2 reports the median MAE and MAPE across seven runs, and Table 3 reports the corresponding IQRs. The results are interpreted as a comparison of the implemented and tuned configurations within the present dataset and evaluation framework rather than proof of universal algorithm superiority.

**Table 2:** Median MAE (min) and MAPE (%) across the four feature sets.

	Median test MAE (min) [MAPE (%)]			
Method	F6	F11	F16	F21
Medium Gaussian SVM	8.57 (81.44)	7.98 (77.05)	8.11 (67.87)	8.27 (68.58)
Boosted Trees	8.33 (88.27)	8.04 (80.15)	8.23 (70.65)	7.94 (69.41)
Quadratic SVM	8.77 (86.36)	8.70 (81.43)	8.57 (71.20)	8.94 (76.40)
Linear SVM	9.24 (91.52)	8.23 (80.02)	8.71 (74.37)	8.74 (75.29)
Efficient Linear SVM	9.32 (92.44)	8.24 (81.05)	8.75 (74.09)	8.80 (76.82)
SVR	8.72 (74.34)	8.67 (84.47)	8.86 (81.48)	8.83 (80.62)
XGBoost	8.38 (85.89)	8.49 (84.64)	8.25 (83.51)	8.39 (81.87)
RF	8.57 (83.58)	8.90 (85.30)	8.49 (79.35)	8.63 (82.42)
LightGBM	8.80 (79.19)	8.62 (84.31)	8.39 (83.78)	8.41 (83.19)
CatBoost	8.48 (88.35)	8.64 (88.31)	8.56 (86.33)	8.56 (85.81)
GBDT	8.29 (85.87)	8.58 (90.37)	8.37 (83.63)	8.52 (82.93)
Bagged Trees	8.57 (92.64)	8.39 (89.24)	8.60 (74.79)	8.17 (72.25)
Matern 5/2 GPR	8.86 (104.83)	8.26 (93.00)	8.54 (85.08)	8.63 (85.02)
Exponential GPR	8.81 (98.62)	8.45 (97.58)	8.58 (86.54)	8.61 (86.56)
Rational Quadratic GPR	8.83 (105.02)	8.27 (93.13)	8.53 (84.95)	8.66 (84.83)
Squared Exponential GPR	8.80 (106.09)	8.27 (93.34)	8.58 (85.62)	8.69 (85.79)
Robust Linear	9.28 (104.30)	8.24 (90.35)	8.67 (80.11)	8.87 (79.62)
Coarse Gaussian SVM	9.25 (95.30)	8.29 (81.91)	8.88 (77.26)	8.65 (81.91)
Medium Tree	8.90 (102.60)	9.07 (93.26)	8.95 (70.93)	8.74 (75.47)
Linear	9.49 (119.67)	8.64 (103.56)	8.85 (89.12)	8.77 (86.99)
Least Squares Regression Kernel	9.29 (110.07)	8.92 (100.07)	8.78 (88.80)	8.69 (87.59)
Fine Tree	9.06 (97.77)	9.15 (87.11)	9.80 (82.00)	9.94 (78.96)
Coarse Tree	8.92 (105.53)	9.01 (98.04)	9.73 (93.28)	9.00 (89.70)
SVM Kernel	9.75 (114.46)	8.80 (99.39)	8.95 (87.11)	9.07 (90.73)
Cubic SVM	9.81 (80.66)	9.21 (90.72)	10.35 (90.32)	9.96 (92.94)
Narrow Neural Network	9.38 (108.17)	9.52 (89.75)	9.69 (94.83)	9.23 (86.21)
Bilayered Neural Network	8.79 (92.91)	9.03 (86.16)	9.69 (94.52)	10.31 (85.22)
Trilayered Neural Network	9.01 (89.56)	9.96 (101.66)	12.46 (91.93)	10.18 (89.65)
Efficient Linear Least Squares	9.50 (119.70)	8.66 (104.05)	8.89 (89.26)	8.74 (87.14)
Fine Gaussian SVM	8.83 (79.73)	10.00 (117.11)	10.40 (125.55)	10.56 (130.70)
Medium Neural Network	8.80 (89.71)	10.79 (109.14)	12.40 (131.39)	12.96 (126.69)
Wide Neural Network	10.13 (95.19)	14.36 (154.18)	15.50 (168.53)	15.00 (144.53)

**Table 3:** Interquartile ranges of MAE (min) and MAPE (%) across the four feature sets.

	IQR of test MAE (min) [MAPE (%)]			
Method	F6	F11	F16	F21
Medium Gaussian SVM	0.67 (15.23)	0.75 (17.09)	0.96 (8.42)	0.26 (7.85)
Boosted Trees	0.73 (19.33)	0.63 (15.00)	0.58 (8.38)	0.42 (7.87)
Quadratic SVM	0.93 (13.46)	0.81 (28.86)	0.86 (10.76)	0.34 (15.44)
Linear SVM	0.48 (13.86)	0.84 (16.90)	0.96 (19.02)	0.51 (11.46)
Efficient Linear SVM	0.58 (11.12)	0.73 (17.08)	0.92 (18.88)	0.50 (12.21)
SVR	0.95 (11.73)	0.96 (22.91)	0.88 (20.44)	0.70 (22.82)
XGBoost	1.00 (18.66)	1.05 (25.55)	0.71 (26.85)	0.75 (29.64)
RF	1.06 (22.30)	0.91 (28.36)	0.88 (29.58)	1.10 (26.52)
LightGBM	1.38 (16.51)	1.15 (24.82)	1.15 (23.95)	0.89 (22.53)
CatBoost	1.03 (20.86)	1.02 (27.59)	1.09 (31.16)	1.08 (30.10)
GBDT	0.93 (18.58)	0.92 (28.30)	0.76 (27.89)	0.88 (27.39)
Bagged Trees	0.65 (17.68)	0.57 (17.24)	0.38 (13.85)	0.12 (12.47)
Matern 5/2 GPR	0.57 (20.92)	1.08 (19.92)	0.65 (13.13)	0.27 (7.53)
Exponential GPR	0.57 (20.63)	0.94 (20.27)	0.68 (14.36)	0.42 (8.97)
Rational Quadratic GPR	0.58 (20.34)	1.09 (19.38)	0.64 (13.36)	0.23 (7.50)
Squared Exponential GPR	0.61 (20.89)	1.10 (20.08)	0.61 (13.50)	0.27 (15.05)
Robust Linear	0.62 (14.16)	0.95 (16.12)	0.95 (15.62)	0.50 (7.33)
Coarse Gaussian SVM	0.71 (11.84)	0.65 (15.45)	0.83 (18.41)	0.75 (13.59)
Medium Tree	0.61 (23.76)	0.79 (16.92)	0.85 (15.48)	0.61 (11.04)
Linear	0.71 (16.77)	0.85 (15.49)	0.70 (14.76)	0.61 (8.80)
Least Squares Regression Kernel	0.91 (18.66)	0.57 (24.30)	0.86 (12.31)	0.50 (8.13)
Fine Tree	0.43 (20.49)	0.40 (12.52)	0.84 (13.36)	0.65 (12.31)
Coarse Tree	0.75 (18.49)	0.67 (11.39)	1.07 (11.25)	0.78 (8.15)
SVM Kernel	0.77 (22.99)	0.56 (23.72)	0.71 (19.05)	0.79 (11.45)
Cubic SVM	1.01 (23.49)	1.77 (26.62)	1.17 (10.89)	0.87 (5.14)
Narrow Neural Network	1.46 (16.37)	0.93 (27.38)	0.75 (9.05)	0.90 (15.67)
Bilayered Neural Network	0.69 (30.23)	1.33 (17.50)	1.43 (17.89)	1.64 (9.25)
Trilayered Neural Network	0.26 (24.84)	1.09 (19.12)	1.46 (26.46)	2.29 (25.67)
Efficient Linear Least Squares	0.70 (16.66)	0.83 (15.51)	0.72 (15.67)	0.59 (9.45)
Fine Gaussian SVM	0.45 (12.76)	0.59 (42.16)	0.91 (19.16)	0.46 (15.45)
Medium Neural Network	0.65 (20.15)	1.18 (20.88)	2.58 (58.56)	2.40 (30.76)
Wide Neural Network	0.91 (10.30)	1.20 (46.94)	0.83 (46.72)	1.98 (29.83)

Among the screened configurations, the Medium Gaussian SVM was consistently competitive. Its median test MAPE decreased from 81.44% in F6 to 67.87% in F16 and remained similar at 68.58% in F21. Boosted Trees and Quadratic SVM were also competitive in the larger feature sets, although their relative performance varied across feature settings.

The IQR results in Table 3 indicate that the Medium Gaussian SVM had comparatively stable repeated-run performance in F16 and F21. Considering both the evaluation metrics and the need for a compact feature set, the tuned F16 Medium Gaussian SVM was selected for subsequent interpretation. This selection does not imply that the algorithm is universally superior outside the present data and evaluation framework.

With respect to feature sets, the results indicate a general improvement in predictive performance from F6 to F16 across many models. However, the additional increase from F16 to F21 does not consistently yield further gains, and in some cases, the improvement is marginal. This pattern suggests that the 16-feature configuration provides a practical balance between predictive accuracy, robustness, and model simplicity.

To provide a classical benchmark, the Medium Gaussian SVM was compared with the linear regression model across all four feature sets. Relative to Linear, the Medium Gaussian SVM reduced MAE by 9.69%, 7.64%, 8.36%, and 5.70% for F6, F11, F16, and F21, respectively, and reduced MAPE by 31.95%, 25.60%, 23.84%, and 21.16%. These reductions consistently favored the nonlinear SVM configuration. Fig 4 presents the mean test MAE and MAPE, together with the corresponding two-sided 95% confidence intervals across the seven repeated runs for the 32 model configurations under the F16 setting.

**Fig 4:**
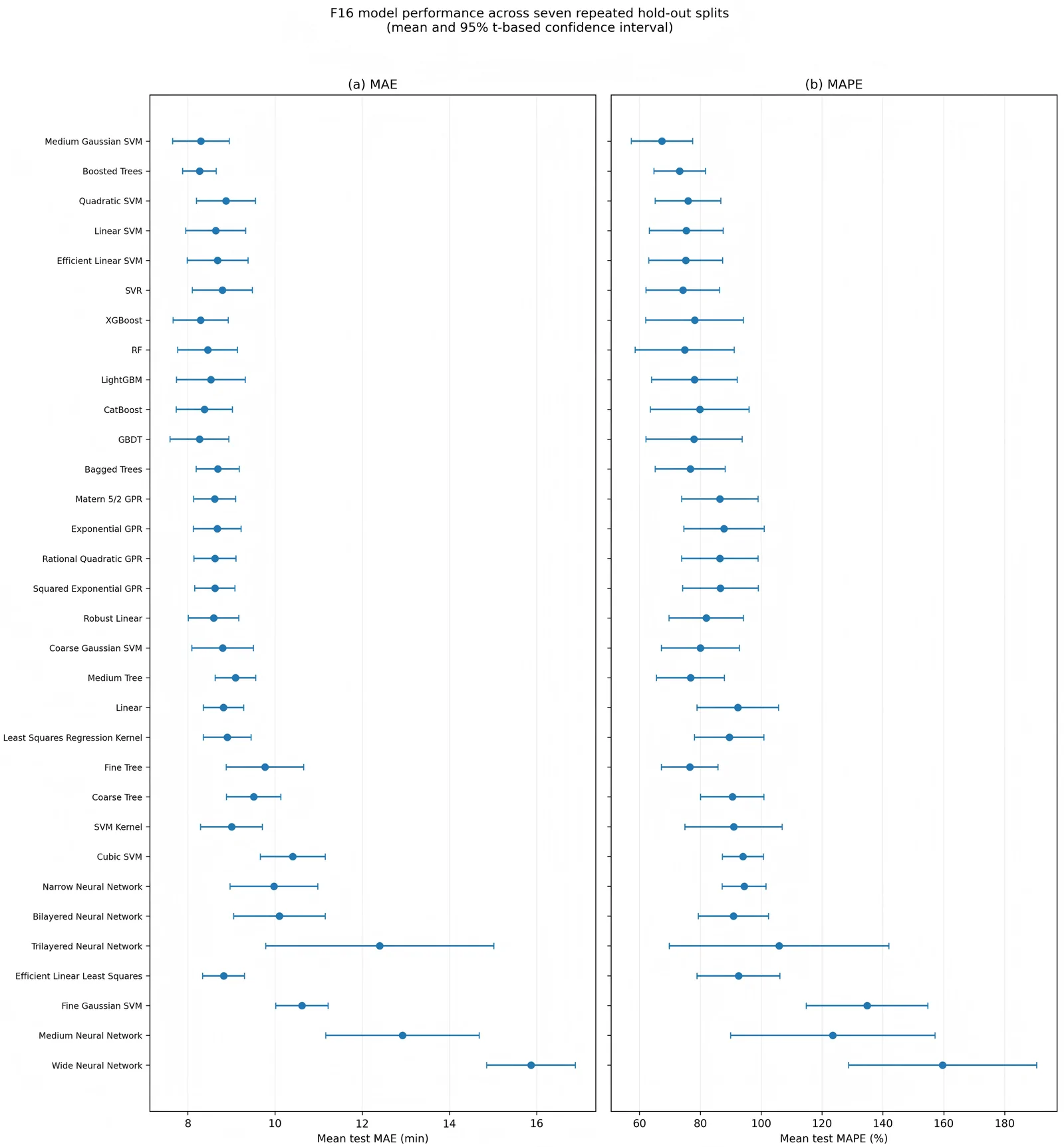
Mean test MAE and MAPE with 95% confidence intervals for F16.

To assess the influence of long-duration incidents, a sensitivity comparison used the 32 F21 model configurations as paired analytical units and contrasted the 911-record complete-case dataset with the 828-record routine-incident dataset (Table 4). Across configurations, mean MAE decreased from 19.44 to 9.23 min and mean MAPE decreased from 119.12% to 87.25% after incidents longer than 60 minutes were excluded. Paired t tests across configurations yielded p < 0.001 for both metrics. These results show that the duration filter materially changes reported performance and support restricting the main conclusions to routine incidents. Because the 911-record results came from an earlier single-run analysis, this comparison is interpreted as a sensitivity check rather than a repeated-run validation.

**Table 4:** Sensitivity of F21 performance to the exclusion of incidents longer than 60 minutes.

Metric	911 records(long-duration incidents retained)	828 records(incidents >60 min excluded)	Paired t-test p-value
Mean MAE (min)	19.44	9.23	2.50 × 10 ^−^ ^27^
Mean MAPE (%)	119.12	87.25	2.76 × 10 ^−^ ^9^

Note: Values represent the mean performance across 32 F21 model configurations.

### Model Explanation

Interpretation analyses were performed for the selected F16R2 Medium Gaussian SVM session. F16R2 was chosen because its hold-out MAPE (67.87%) matched the seven-run median for the F16 configuration, thereby avoiding selection of a best-case run. Local Shapley values describe contributions to individual predictions, whereas the mean absolute Shapley values summarize global importance across the 200 query points. The partial-dependence, residual, and validation plots were generated from the same saved session.

### Global Shapley Importance

Fig 5 presents the mean absolute Shapley values for the F16R2 model. Response Time has the largest global importance, followed by Hard Shoulder Blocked, Number of Vehicles, and Weather. Because Response Time is a component of the incident timeline and is available only after arrival, its prominence should be interpreted as evidence for post-arrival duration updating rather than as an exogenous causal effect. Alarm Source, PC involvement, Section, Weekend, lane-specific blockage, HV involvement, Injury Involvement, Incident Severity, and Vehicle Breakdown show smaller marginal contributions in this model.

**Fig 5:**
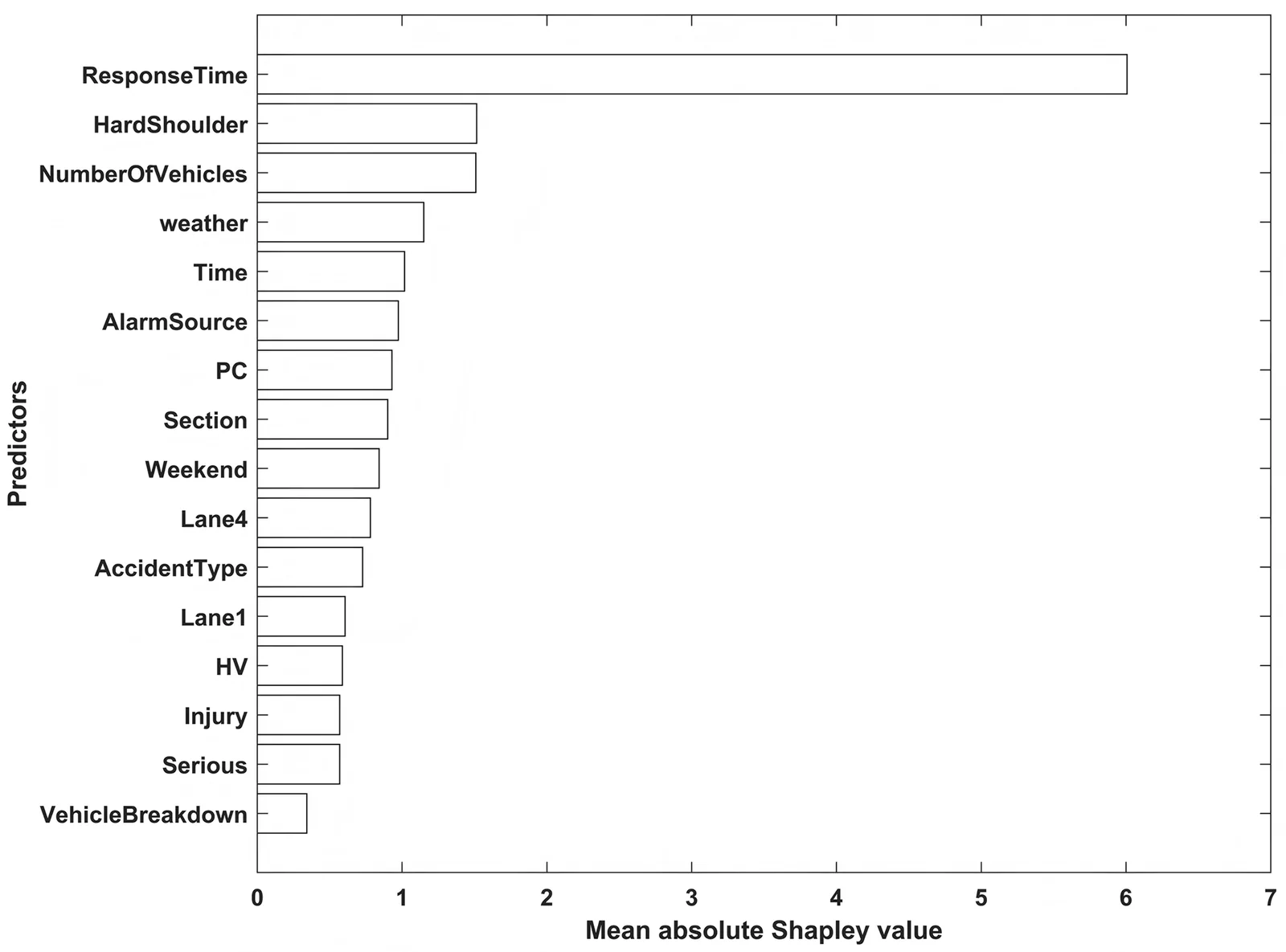
Mean absolute Shapley values.

### Global Partial Dependence

Global partial dependence plots were used to examine the marginal relationship between selected predictors and the predicted incident duration. These plots show how the model output changes as one predictor varies while averaging over the remaining predictors. To save space, only the top four contributors are shown here. Fig 6 presents the four most influential predictors identified by the Shapley analysis: response time, hard shoulder blockage, number of vehicles, and weather.

1. Response time shows a clear positive association with predicted duration (Fig 6a). As response time increases, the predicted incident duration rises substantially, although the slope becomes flatter at higher values. This pattern indicates that delayed responder arrival is strongly associated with longer incident resolution time.
2. Hard shoulder blockage produces a distinct categorical difference (Fig 6b). Incidents with hard shoulder blockage are associated with higher predicted durations than those without blockage. This result is operationally plausible because shoulder blockage reduces maneuvering space, constrains temporary traffic organization, and may complicate emergency response and vehicle clearance.
3. The number of vehicles also affects the predicted duration (Fig 6c). The predicted duration is lower for the middle category and higher for the other categories, suggesting that incident complexity is not determined solely by vehicle count, but also by the composition and operational characteristics of the involved vehicles.
4. Weather shows categorical differences in predicted duration (Fig 6d), indicating that environmental conditions influence model output. Adverse weather is associated with longer predicted durations, which is consistent with reduced visibility, lower operating speeds, and more difficult on-site handling under unfavorable conditions.

**Fig 6:**
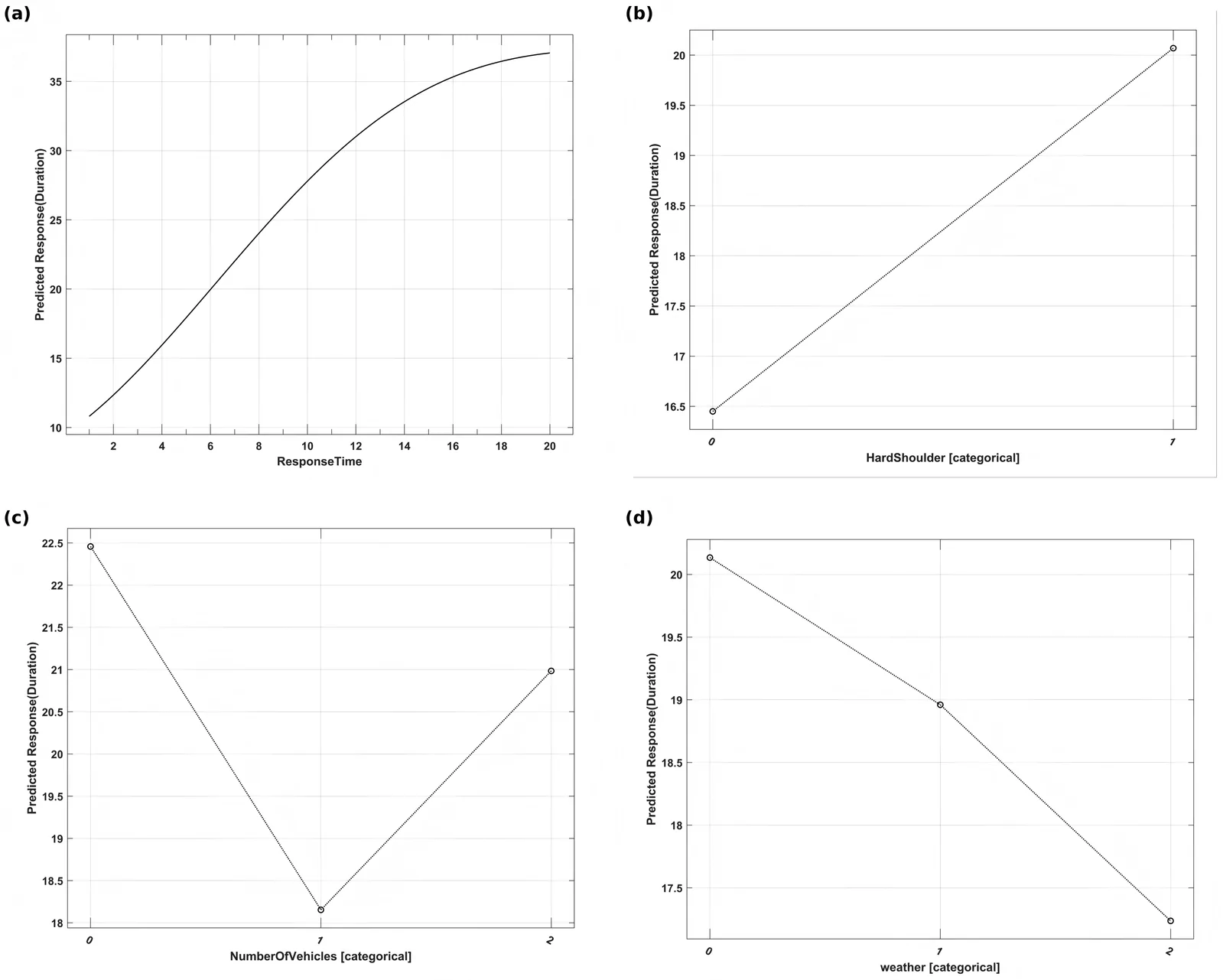
Global partial dependence plots for (a) Response Time, (b) Hard Shoulder Blocked, (c) Number of Vehicles, and (d) Weather.

### Residual Plots

Fig 7 illustrates the residual behavior of the selected Medium Gaussian SVM model. Fig 7a, 7b, and 7c show observations versus predictions, observations versus residuals, and predictions versus residuals, respectively. As shown in Fig 7a, the model captures the general relationship between observed and predicted duration, but the deviation from the perfect-prediction line increases as incident duration becomes larger. Fig 7b indicates that residuals are concentrated near zero for shorter incidents, whereas positive residuals become larger and more frequent as the observed duration increases, suggesting systematic underestimation for longer incidents. Fig 7c further shows that the spread of residuals becomes wider at higher predicted durations. Overall, the Medium Gaussian SVM model performs reasonably well for routine incidents, but its prediction errors increase for longer and more complex events. Therefore, predictions in the upper duration range should be interpreted with caution.

**Fig 7:**
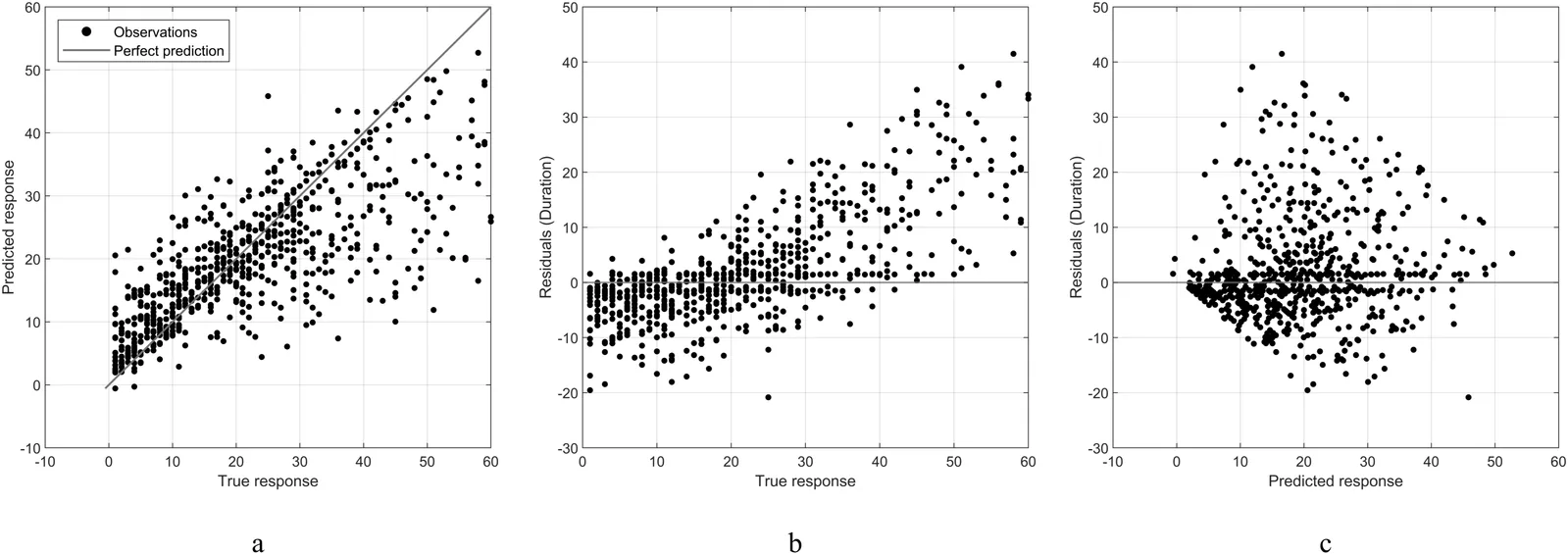
Diagnostic plots: (a) Observations vs. Predictions, (b) Observations vs. Residuals, and (c) Predictions vs. Residuals.

### Validation Plots

To further assess the agreement between model predictions and observed data, Figs. A1–A16 are provided in the Supporting Information. For the 15 categorical predictors, boxplots are used to compare the observed and predicted duration distributions across categories. For the continuous predictor Response Time, the corresponding validation plot presents the observed values together with their prediction errors. Because the incident duration is strongly right-skewed, the boxplots are interpreted mainly in terms of medians and interquartile ranges rather than means alone.

The validation plots show that accident-related variables produce clear and meaningful differences in incident duration. In the Accident Type plot, noticeable variation is observed across categories. For example, Accident Type = 0 (n = 603) has an observed median duration of 18 min (IQR = 21), whereas Accident Type = 1 (n = 169) has a higher observed median of 24 min (IQR = 15). Accident Type = 2 (n = 46) shows a much lower median duration of 6.5 min (IQR = 9). For the rare categories, Accident Type = 3 (n = 4) and Accident Type = 4 (n = 6) have observed medians of 32 and 45.5 min, respectively, indicating substantially longer durations. The predicted medians preserve the same general ordering, but the higher-duration categories are estimated more conservatively, with predicted medians of 23.01 and 28.75 min for Accident Type = 3 and 4, respectively.

Severity-related variables also display clear patterns. In the Incident Severity plot, the severe category (Incident Severity = 0; n = 24) has an observed median duration of 44.5 min (IQR = 22.5), whereas the non-severe category (Incident Severity = 1; n = 804) has a median of 19 min (IQR = 20). The corresponding predicted medians are 39.99 and 17.97 min, respectively, suggesting that the model captures the directional effect of severity while producing a more compressed distribution. Similar patterns are visible in the HV and PC plots. Incidents involving heavy vehicles (HV = 1; n = 237) have an observed median duration of 22 min (IQR = 26), compared with 19 min (IQR = 19) for HV = 0 (n = 591). When no private car is involved (PC = 0; n = 105), the observed median duration increases to 29 min (IQR = 27), compared with 18 min (IQR = 19) for PC = 1 (n = 723). In all of these cases, the predicted distributions broadly follow the observed direction of change.

Operational and roadway-condition variables provide additional support for the model interpretation. In the Hard Shoulder Blocked plot, the blocked category (Hard Shoulder Blocked = 1; n = 585) has an observed median duration of 22 min, compared with 12 min for the unblocked category (Hard Shoulder Blocked = 0; n = 243). The predicted medians are 19.57 and 11.53 min, respectively, preserving the same directional contrast. For Lane 4, the blocked category (Lane 4 = 1; n = 309) has a higher observed median duration of 23 min than the unblocked category (17 min for Lane 4 = 0; n = 519), and the predicted medians show a similar pattern (21.97 vs. 16.03 min). Across roadway sections, the observed medians range from 15 to 23 min, with Section = 2 showing the highest observed median duration of 23 min, while the predicted distributions broadly follow the same relative ranking.

Temporal, environmental, and detection-related variables also show reasonable trends. In the Time of Day plot, Time of Day = 3 has an observed median duration of 25 min, higher than the 17 min observed for Time = 1, while the predicted medians are 22.69 and 16.06 min, respectively. In the Alarm Source plot, Alarm Source = 1 has the highest observed median duration of 23 min, compared with 16 min for both categories 0 and 2. The predicted medians remain consistent with this ordering. In the Weather plot, Weather = 1 shows the highest observed median duration of 22.5 min, whereas Weather = 0 and 2 have observed medians of 18 and 20 min, respectively. The Weekend plot indicates only modest differences, with observed medians of 19 min for weekdays and 20 min for weekends, which is also reflected in the predicted values.

Finally, the continuous predictor Response Time plays an important role. In the filtered dataset of 828 incidents, response time ranges from 1 to 30 min, with a median of 5 min, a mean of 5.8 min, and a standard deviation of 4.3 min; note that 83% of the incidents have response times below 10 min. The validation plot shows that most incidents are concentrated in the lower response-time range, whereas larger durations and larger prediction errors appear more frequently as response time increases. This is consistent with the positive association observed in Fig. 1, where the Spearman correlation between response time and incident duration is 0.51.

## Discussion and policy implications

### Key findings

Within the current dataset and evaluation framework, the tuned F16 Medium Gaussian SVM provided a favorable balance of predictive accuracy, repeated-run stability, and feature-set size. Performance generally improved from F6 to F16, while the additional variables in F21 provided little further benefit. Relative to classical linear regression, the Medium Gaussian SVM produced lower MAE and MAPE in every feature setting, with MAE reductions of 5.7%–9.7% and MAPE reductions of 21.2%–31.9%. The duration-threshold sensitivity analysis also showed substantially higher errors when prolonged incidents were retained, underscoring that data-cleaning decisions and target-population definition materially affect apparent model performance. The MRMR and explanation results highlight Response Time and several operational or severity-related variables, but these findings reflect predictive associations within a post-arrival assessment setting. The configuration-level benchmark should not be interpreted as evidence that one algorithm will remain best across other corridors, time periods, or implementation choices.

At the same time, the selected Medium Gaussian SVM model becomes less accurate for longer and more complex incidents. The predicted distributions are generally more compressed than the observed ones, and the residual analysis indicates systematic underestimation in the upper duration range. MAE and MAPE were intentionally retained as the primary evaluation metrics because they provide directly interpretable measures of absolute and relative prediction error. Reporting the relatively high MAPE values allows the limitations of the models to be presented transparently and avoids overstating their operational performance. Although MSE and RMSE characterize prediction errors from a different perspective, relying on them alone could obscure the substantial percentage errors observed for short-duration incidents. Therefore, MAPE should be interpreted jointly with MAE rather than in isolation. The higher errors observed when incidents longer than 60 minutes were retained further indicate that the current predictors do not fully capture the operational processes behind prolonged events. Therefore, the model should be interpreted primarily as a decision-support tool for routine incidents rather than a high-reliability predictor for rare extreme events.

### Policy Recommendations

Based on these findings, the following policies and operational strategies are suggested to improve incident response and reduce clearance times:

1. Shorter response times were associated with shorter incident durations in the current dataset. Measures that improve incident detection, dispatch efficiency, and inter-agency coordination may therefore contribute to shorter clearance times, although the present predictive analysis does not establish a causal effect.
2. Incidents associated with higher operational complexity, such as rollover crashes, heavy-vehicle involvement, multiple-vehicle crashes, and shoulder or lane blockage, should receive priority in subsequent resource escalation and clearance planning. After the initial responder arrival, updated duration estimates may support the additional deployment of towing equipment, traffic-control resources, and specialized response personnel.
3. The results indicate that useful decision-support performance does not necessarily require the largest feature set. Since the improvement beyond the 16-feature setting is limited, traffic agencies may focus on collecting a compact group of highly informative variables rather than maximizing data quantity alone.
4. Because longer and more complex incidents remain more difficult to predict, future operational systems should incorporate additional process-oriented information, such as towing timelines, lane-specific closure duration, secondary crashes, or on-site investigation details. Such information may improve prediction for difficult cases that are currently underestimated by the model.

## Conclusions

This study compared 32 machine-learning model configurations under four fixed MRMR-ranked feature sets. All 32 models were tuned using sevenfold cross-validation within the training subset, and the tuning procedure and optimal parameters of the selected Medium Gaussian SVM were reported as a representative example. The repeated 15% hold-out design and sevenfold cross-validation showed that F16 offered a practical balance between predictive performance and feature-set size. The selected SVM also produced consistently lower MAE and MAPE than classical linear regression across the four feature settings. The sensitivity comparison confirmed that retaining incidents longer than 60 minutes substantially increased reported errors. The results therefore support cautious post-arrival duration updating for routine incidents, not deterministic prediction at the initial alarm stage or for prolonged high-impact events.

Across the MRMR ranking, Shapley analysis, and category-level validation, Response Time was consistently influential, while Injury Involvement, Hard Shoulder Blocked, Accident Type, Number of Vehicles, and Weather also showed important predictive or category-level patterns. These findings represent predictive associations rather than causal effects. The validation analysis further showed that the selected Medium Gaussian SVM reproduced the main category-level differences in the data. More severe incidents, hard-shoulder blockage, heavy-vehicle involvement, and the absence of private-car involvement were associated with longer observed durations, and the predicted distributions broadly preserved these directional patterns. However, the predicted distributions were generally more compressed than the observed distributions, indicating that the model smoothed some extreme cases.

Despite its findings, this study has three main limitations. (1) The data were collected from a single freeway corridor during one year, and chronological and external validation were not available. The transferability of the findings to other corridors, time periods, and operational environments therefore remains to be established. (2) The four feature sets were defined from an MRMR ranking calculated once before the repeated train–test splits rather than recalculated within each training subset. Therefore, some feature-selection optimism cannot be completely excluded. In addition, formal statistical significance tests across all model configurations were not conducted. Future studies should incorporate feature selection within each training split and use formal paired model-comparison procedures. (3) Long-duration incidents and rare severe categories remain difficult to predict because the available structured data did not fully capture the operational processes associated with complex events. The 911-record sensitivity results were retained from an earlier single-run analysis and should therefore be interpreted as a scope check. Additional variables describing towing progress, lane-closure evolution, secondary events, and on-site investigation activities may improve prediction performance for these cases.

Future research should use multi-corridor and multi-year datasets, chronological and external validation, nested feature selection, and formal paired model-comparison procedures based on retained run-level predictions. Fully reproducible pipelines should retain run-level seeds, preprocessing objects, parameter grids, quartiles, predictions, and final fitted models. Separate or staged models may be appropriate for initial-alarm prediction, post-arrival updating, routine incidents, and prolonged high-impact incidents. Additional variables describing towing, lane-closure progression, traffic states, secondary events, and investigation activities may improve performance for difficult cases.

## Supporting information

S1 Fig Accident Type. (TIF)

S2 Fig Time of Day. (TIF)

S3 Fig Weekend. (TIF)

S4 Fig Alarm Source. (TIF)

S5 Fig Lane 1. (TIF)

S6 Fig Lane 4. (TIF)

S7 Fig Hard Shoulder Blocked. (TIF)

S8 Fig Section. (TIF)

S9 Fig PC. (TIF)

S10 Fig HV. (TIF)

S11 Fig Injury Involvement. (TIF)

S12 Fig Incident Severity. (TIF)

S13 Fig Vehicle Breakdown. (TIF)

S14 Fig Number of Vehicles. (TIF)

S15 Fig Weather. (TIF)

S16 Fig Response Time. (TIF)
